# Digital twins as self-models for intelligent structures

**DOI:** 10.1038/s41598-025-14347-8

**Published:** 2025-08-19

**Authors:** Xiaoxue Shen, David J. Wagg, Matthew Tipuric, Matthew S. Bonney

**Affiliations:** 1 The Alan Turing Institute, London, NW1 2DB UK; 2 School of Mechanical, Aerospace and Civil Engineering, University of Sheffield, Sheffield, S1 3JD UK; 3 School of Aerospace, Civil, Electrical and Mechanical Engineering, Swansea University, Swansea, SA1 8EN UK

**Keywords:** Digital twin, Self-model, Agent, Structure, Engineering, Civil engineering, Mechanical engineering

## Abstract

A self-model is an artificial intelligence that is able to create a continuously updated internal representation of itself. In this paper we use an agent-based architecture to create a ‘digital twin self-model’, using the example of a small-scale three-story building. The architecture is based on a set of heterogeneous digital components, each managed by an agent. The agents can be orchestrated to perform a specific workflow, or collaborate with a human user to perform requested tasks. The digital twin architecture enables multiple complex behaviors to be represented via a time-evolving dynamic assembly of the digital components, that also includes the encoding of a self-model in a knowledge graph as well as producing quantitative outputs. Four operational modes are defined for the digital twin and the example shown here demonstrates an offline mode that executes a predefined workflow with five agents. The digital twin has an information management system which is coordinated using a dynamic knowledge graph that encodes the self-model. Users can visualize the knowledge graph via a web-based user interface and also input natural language queries. Retrieval augmented generation is used to give a response to the queries using both the local knowledge graph and a large language model.

## Introduction

In this paper, we consider how a digital twin might be used as a *self-model* in the context of structural engineering. The motivation is to move towards the idea of so called ‘intelligent’ (or ‘smart’) structures — something that has been an aspiration of engineers since the 1980s^1^. Up until now ‘low-level’ forms of intelligence have been developed, based on using material and control design (and more recently machine learning) to enable structures, such as buildings, bridges, or aircraft, to sense and respond to their environments^2–5^. In this paper we consider a different aspect of intelligence applied to structural engineering, that of the *self-model*^6^. More specifically, we argue that a digital twin can be used as a *self-model* for an intelligent structure.

Self-modeling is the process whereby an intelligent agent learns to model its own time evolving behavior. Self-modeling occurs in intelligent lifeforms (e.g. in humans and animals) and is a desirable attribute for entities with artificial intelligence (AI), such as robots^7^. We will define a self-model as an artificial intelligence capable of creating a continuously updated internal representation of itself. In this context, we propose to use a digital twin as the self-model for an intelligent structure.

Digital twins are virtual representations of a physical object or process called the physical twin, with the capacity for bi-directional data-exchange between the digital and physical domains^8^. In this work we will make an explicit distinction between the physical twin (we use a small-scale building as the example) and its surrounding physical environment^9^. The digital twin will be developed as a self-model of the physical twin, and the surrounding physical environment can then be considered either as a world-model^10^ in which the self-model operates or (as here) simply as an assumed boundary with the self-model. We therefore create a world-self-model system^9^, where the digital twin acts as the self-model. Our approach is distinct from previous work which typically considers digital twins as creating a complete interconnected virtual representation of the physical world, such as the ‘world avatar’ concept^11–13^.

To demonstrate an initial version of the concept, we focus on creating a self-model that is an informational representation consisting of ‘self-knowledge’ and ‘memory’. The self-knowledge and memory are contained in an information management system^14–17^ that allows the digital twin to accumulate knowledge and also respond to queries from human users and agents. In this paper, the information management system for the digital twin is coordinated using a knowledge graph that is implemented using the Neo4j graph database package^18^. On first launch of the digital twin, the knowledge graph is built based on an initially available set of information. Following that, subsequent actions taken by the agents (or human users) are recorded as events in a dynamically updated version of the knowledge graph. In this way, the self-model is encoded in the knowledge graph.

### Choice of example structure

Building structures form part of the built environment, and are often surrounded by many other similar structures in urban areas and cities^19,20^. Buildings are also complex systems in their own right, requiring increasingly advanced forms of asset management to optimize functions such as energy use based on human occupation^21^. Here we use a small-scale building as the example physical twin, because the system can be complex in its own right, but also part of a bigger more complex system^22^. Thus, we make a distinction between the self-model of the building itself, and a world-model of the environment surrounding the building.

To charactierize the level of intelligence, we adopt a four-level system where Level-0 means no intelligence, and Levels 1–3 progressively introduce aspects of intelligence^9^. In this work, the most important aspects of Level 1–3 intelligence are that the digital twin (i) has a goal, (ii) can receive, process & output information to pursue the goal, (iii) has an informational representation of itself & the world, (iv) can self-update, (v) can abstract information from physical reality & transfer information into physical actions, and (vi) is able to interact with the physical world.

In order to demonstrate the concept, we will focus on aspect (iii), which is creating an informational representation of itself & the world. In our study the informational representation is limited to a system of self-knowledge about the constituent parts and behaviors of the structure including ‘memory’ of events that occur during a prescribed timeline. To achieve this, we use an architecture based on a set of ‘components’, each managed by an intelligent agent that can perceive information (across a communication network) and respond by taking actions. Each of the agents has responsibility for curating a component, and by orchestrating the agents together the components can be ‘assembled’ into a digital twin^23^.

The approach we take here is that the entire architecture of the digital twin is designed as a network of collaborating agents to enable both the dynamic assembly of components and the construction of a self-model which can maintain an updated internal representation of itself. Sample results are presented in the Results Section to show how the overall process works for the example system.

### Related work

Self-models have a long history of development in cognitive science and philosophy^24^ and more recently in AI and robotics^6,7^. In a different context from this work, agent-based models within digital twins have already been considered by several authors, typically by using agent-based modeling to capture some process of interest within the digital twin^19,25–28^. More broadly, intelligent agents are an important tool in AI applications^29–34^. There are multiple interpretations and definitions of ‘agent’ including the rapidly developing topic of agentic systems where, for example, large language models (LLMs) are used as the agents^35^. Other approaches to building digital twins using graphical models, have also been developed^36,37^.

There is a wide literature relating to knowledge modeling^38–41^ for AI systems. Both knowledge graphs and LLMs have been developed extensively in the domain^12,42,43^. Digital twins including knowledge graphs have been developed, for example as part of the ‘world avatar’ concept^11–13^.

We note that there is no single definition of intelligence in the context of AI^44^, so here we adopt a four-level system previously described in the context of world-self-models^9^.

**Fig. 1:**
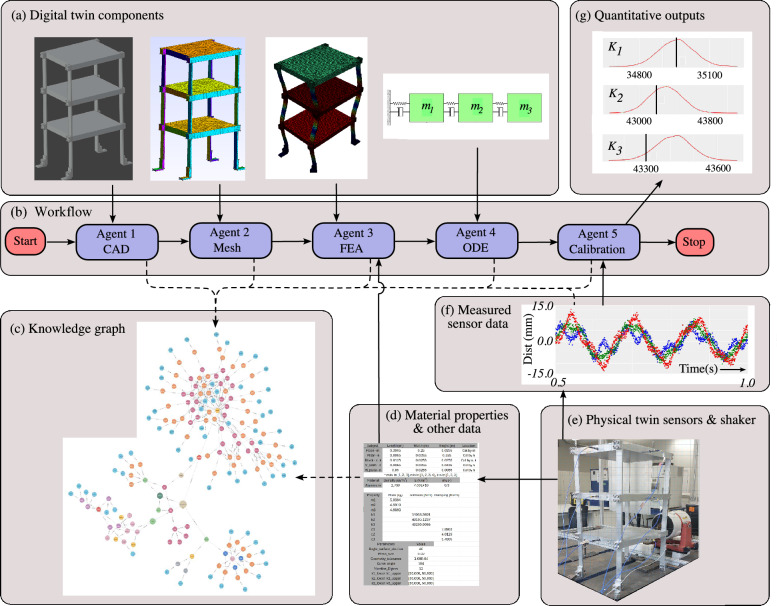
Creating a digital twin (DT) self-model from a predefined workflow for the three-story building example, showing **a** the DT components, **b** the predefined workflow, **c** the knowledge graph which encodes the self-model, **d** additional data sources, **e** the physical twin (PT), **f** a sample of the measured data from the PT, and (g) quantitative outputs. The solid arrows show initial data flows and the dashed lines show dynamically updated data/information flows into the knowledge graph.

## Results

### Agent-based workflow

A sample result of creating the digital twin self-model for the three-story building example is shown in Fig. 1. In this example, a predefined workflow (Fig. 1b) has been designed for the three-story building digital twin. The workflow has a sequential structure, and will be started by the human user, after which there are a sequence of five requests to Agents 1 to 5. Each of the digital twin components (Fig. 1a) in this example are managed by Agents 1 to 5.

Agents 1 and 2 are designed to deal with the geometric complexity in this particular example. The information generated by Agents 1 & 2 is then passed onto the knowledge graph (Fig. 1c) and Agent 3 which deals with an aspect of the behavioral complexity for this example. Agent 3 also requires additional inputs that define the material properties, boundary conditions (eg the interface with the world model) and other details of the physical twin. This is provided using tabulated information based on the design and manufacture of the building (Fig. 1d). Outputs from Agent 3 include parameter matrices than can be used by Agents 4 and 5 which ultimately create the required quantitative and visual outputs that can be returned to the human user (Fig. 1g). Further details of the workflow, including architecture and implementation, are given in the Methods Section.

### Encoding the self-model within the knowledge graph

The time-evolving knowledge graph is initially seeded from the available information (Fig. 1d) to represent the initial parameters and inputs of the building structure. As the workflow proceeds, the knowledge graph is updated in parallel (dashed lines in Fig. 1) to record the outputs from each Agent as entities and relationships in the knowledge graph. Seeding the knowledge graph requires context specific inputs from the digital twin designer (or a human user), which includes the initial computer-aided-design (CAD) geometry, and tabulated information about the material properties, joints, sensors, actuators & loads (often collated into a ‘bill of materials’), other boundary conditions (eg the interface with the world model) including parameters such as temperature etc. After seeding, and as the workflow progresses, each time an agent performs a task it also triggers an update to the knowledge graph, which evolves dynamically as the workflow progresses. The knowledge graph therefore essentially encodes the self-model, meaning it contains the knowledge representation of the physical twin and the associated computational agents, which can then be used as part of a query system for human users.

### User query results

**Fig. 2:**
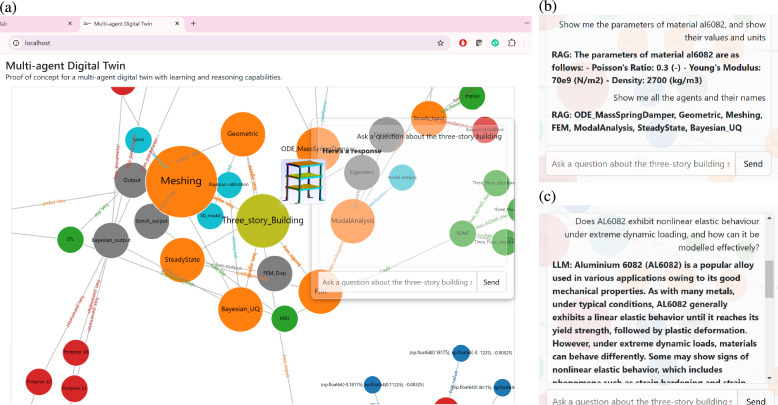
Screenshots of the user interface of the three-story building digital twin. **a** The web-page that forms the user-interface in this example, where a visualization of the knowledge graph is shown and the user can also input queries. Note that when the user’s cursor hovers over the agent node, the node dynamically enlarges, and the output generated by the agent is displayed adjacent to it (in this case the Gmsh image). This output visualization persists only during the hover interaction and is concealed once the cursor moves away from the node. The query system uses a form of retrieval augmented generation (RAG) to combine the local knowledge graph with a large language model (LLM). **b** A query which is answered using information from the local knowledge graph (the cursor response is RAG). **c** A query which is answered using information from the LLM (cursor shows as LLM).

In terms of what the human user experiences, an example user interface is shown in Fig. 2a, which in this case is a web browser window. The user interface can be customized to suit the particular application of the digital twin. In this case the user interface is designed as a proof-of-concept that showcases just a small number of desirable functionalities. For example, the interface shown on the left of Fig. 2 can be used to visualize the knowledge graph and interact with a chatbot-like query system.

The chat interface allows users to send questions via a text input field and receive responses from a server endpoint. Answers to the chat interface are provided by a combined knowledge graph and language model (LLM)-based question-answering system. The combination of knowledge graph and LLM is a form of retrieval augmented generation (RAG) that sends the user query to both the local knowledge graph and the LLM (which in this case is Chat-GPT^43^).

Examples of a human user making queries via the query box in the user interface are shown on the right side of Fig. 2. Figure 2b illustrates the results obtained when querying information explicitly available within the knowledge graph, such as the properties of material AL6082, the agents in the digital twin and their respective names. Conversely, Fig. 2c illustrates the outcome of queries for information that is absent from the local knowledge graph. In such cases, the query system switches to the external LLM API to retrieve data from internet-based sources, ensuring the provision of comprehensive responses when local graph data are incomplete or unavailable. The user interface indicates which system is answering by prefixing the response with ‘RAG:’ when the local knowledge graph responds and ‘LLM:’ when the LLM API responds.

This example is designed to demonstrate how a self-model can be used to enable increased intelligence (Level-1 in this case^9^) in a digital twin of a structure. Specifically, the goal was to build a system of self-knowledge (e.g., the knowledge graph within the IMS) that can respond to natural language queries accurately and efficiently via RAG^45,46^.

**Fig. 3:**
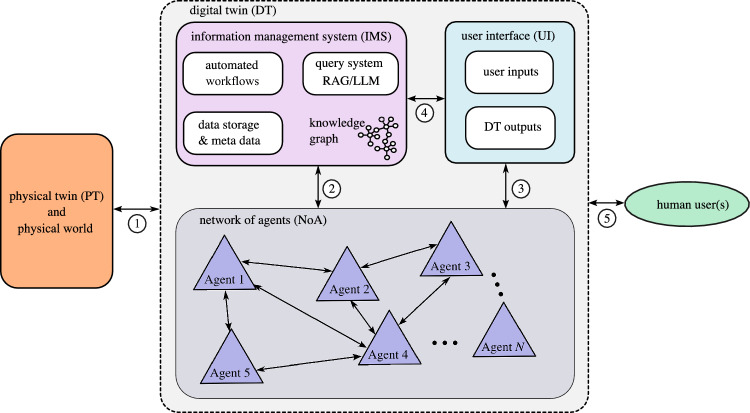
The overall architecture of the digital twin. *N*-agents are used to perform specific computational tasks within the digital twin. The agents communicate across a network. The information management system is coordinated with a dynamic knowledge graph, and the user interface allows the user to interact with the digital twin. The physical twin has local edge hardware such that data can be collected, and control actions taken to adjust the behavior of the physical twin. The numbered circles denote the main points of information exchange. Specifically $\textcircled {1}$ is the data exchange between the physical and digital twin, $\textcircled {2}$ is the exchange between the *N* agents and the IMS, $\textcircled {3}$ is the UI interface with the agents, $\textcircled {4}$ the UI and IMS and finally $\textcircled {5}$ is the human user interaction with the digital twin.

## Methods

### Digital twin architecture and implementation

The overall architecture of the digital twin is shown schematically in Fig. 3. In this architecture, the digital twin is composed of three main parts that enable a connection between the physical twin and the human user(s). Firstly, there is a user interface through which human users give inputs and view the outputs of the digital twin, as shown in Fig. 2. The user interface directs the input to and outputs from the two other main parts. These parts are, firstly a network of *N* collaborative agents which are used to perform specific tasks within the digital twin. Each agent is responsible for ‘curating’ a specific component in the digital twin, where curating in this context means maintaining and updating the component. The agents communicate across a network, and each agent has a set of potential actions it can take, depending on the task at hand. All actions across the network are logged, and recorded as events in the temporal part of the information management system (IMS) which is coordinated using the knowledge graph. The numbered circles in Fig. 3 denote the main points of information exchange.

**Fig. 4:**
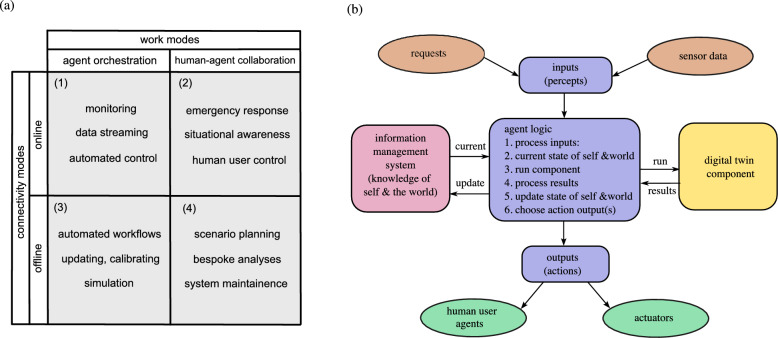
**a** Modes of operation for the digital twin as a combination of the connectivity modes and work modes. The cases are (1) online mode with agent orchestration, (2) online mode with the human user and agents collaborating, (3) offline mode with agent-based orchestration, and (4) offline mode with the human user and agents collaborating. **b** A schematic diagram of the structure of an intelligent agent within the digital twin.

In the current context, it is important to understand the possible *modes of operation* for the digital twin, which are shown schematically in Fig. 4a. In this case, the modes of operation for the digital twin are a combination of the ‘connectivity modes’ and ‘work modes’. Note that ‘online’ means the digital and physical twins are actively connected over a network (via data flow $\textcircled {1}$ in Fig. 3), whereas ‘offline’ means that there is not an active connection between physical and digital twins. During the offline mode data flows are still possible via $\textcircled {2}$, $\textcircled {3}$, $\textcircled {4}$ and $\textcircled {5}$ shown in Fig. 3. The cases shown in in Fig. 4a are:

1. *online mode with agent orchestration*; e.g. to carry out automated processes related to monitoring and control of the physical twin,
2. *online mode with the human user and agents collaborating*; e.g. to respond to urgent situations and take command of the situation,
3. *offline mode with agent-based orchestration*; e.g. to carry out predefined background processes and workflows, and
4. *offline mode with the human user and agents collaborating*; e.g. to carry out bespoke analyses and maintenance that isn’t in a predefined workflow.

Normally agents are considered to be taking actions into an ‘environment’ and then learning from the feedback from the action taken. In the architecture proposed in Fig. 3, the agent will only be able to take actions that influence the physical twin (e.g. the physical ‘environment’) during the online connectivity modes. In offline mode, the agents will interact with either prerecorded and/or simulated (e.g synthetic) data. In other words, the agents interact with a virtual environment^47^ only when in offline mode. The example chosen here falls into mode (3), it is an offline mode with a pre-defined, agent orchestrated workflow.

To create a digital twin self model the agents we use need to have some level of intelligence^9^. The specific details of the agents used in this work are considered next.

### Agents

The detailed layout of the agent structure used here is shown schematically in Fig. 4b. The *n*th agent structure can be represented as a tuple $\alpha _n=\langle P, S, C, A \rangle$ with *P* inputs, *S* agent states, *C* component states, and *A* output actions. The agent receives inputs from human users and/or other agents in addition to sensor data across the network $P=\langle p_1, p_2...,p_n \rangle$. Then the agent uses predefined logic to make a decision on what corresponding sequence of output actions is required $A=\langle a_1, a_2...,a_n \rangle$. The decision making process iterates the agent states $S=\langle s_1, s_2...,s_n \rangle$ and relevant DT component states $C=\langle c_1, c_2...,c_n \rangle$.

Each agent is given responsibility for curating (e.g. managing and maintaining) a specific digital twin component. These components are a heterogeneous set of ‘digital objects’^23^ that relate to the specific context of the digital twin, as shown in Fig. 1a. In terms of software architecture, each agent manages a digital object that is located in a software container. Containers are widely used in software development, and have also been using in building digital twins^11^. The container network is used to communicate between agents.

To demonstrate the implementation and potential use of agents, the example case in Fig. 1 will be explained further in this section. The workflow in Fig. 1 proceeds as follows. Agent 1 is used to build/upload the computer-aided design (CAD) files representing the geometric complexity of the building, considered to be input $p_1$. The output from Agent 1 is a set of STL (stereolithography CAD^48^) files of the individual components, as well as the assembled building structure. Each STL file describes a raw, unstructured triangulated surface by the unit normal and vertices of the triangles using a three-dimensional Cartesian coordinate system. For this demonstration, we utilize Blender, an open-source CAD software tool; however, STL is a widely used standardized format across most CAD software tools.

**Fig. 5:**
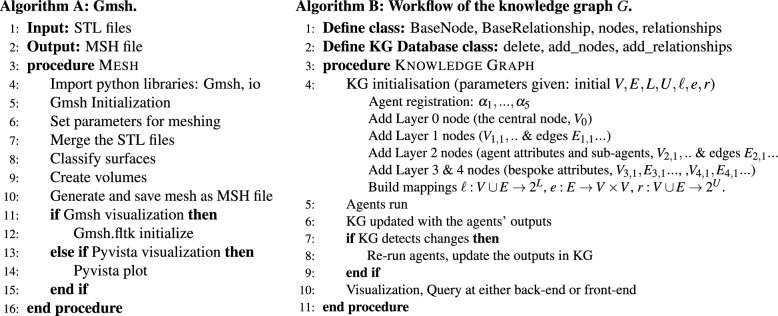
Sample pseudo-code for the algorithms for parts of the agent process. Shown here are: **A** Gmsh meshing process, and **B** workflow of the knowledge graph.

The STL files serve both as the output of the Agent 1 and as the input of the Agent 2, in the form of a mapping $a_1 \rightarrow p_2$. The process carried out by Agent 2 using the Python library Gmsh^49^ is outlined in Algorithm A shown in Figure 5. After initializing Gmsh and setting the meshing parameters, the system merges the STL files stored in the digital twin system. The geometric information is then classified to redefine the surfaces, followed by the creation of discrete curves and surfaces in the mesh. Once these surfaces are created, a volume defined by these surfaces will be prepared for meshing with the specified meshing parameters. For simple geometries, this process can be fully automated.

Finally, Agent 2 converts the mesh to XDMF^50^ format so that it can be recognized by the open-source FEA tool FEniCSx^51^. This process first determines the topological dimension of the existing mesh, the types of cells and facets are defined. These are then extracted from the structured information and reconstructed as a MeshIO mesh in the mapping $a_2 \rightarrow p_3$.

In addition to the mesh, the boundary conditions (type and coordinates), mesh specifications (size), and material properties (density, Young’s modulus, and Poisson’s ratio) are also required as the inputs for Agent 3 (e.g. Fig. 1d) which manages FEniCSx, the finite element analysis (FEA) component. Agent 3 captures the behavioral complexity via two types of analysis: modal analysis and steady-state analysis. In this modal analysis case, the eigenfrequencies and mode shapes are compared with the data from the physical twin. The results of Agent 3 are then mapped to the input of Agent 4, $a_3 \rightarrow p_4$ where they are verified against a reduced order ordinary differential equation model^8^. Lastly, with mapping $a_4 \rightarrow p_5$, Agent 5 performs Bayesian calibration^52^ that can calibrate the model parameters of Agent 4, using data measured from the physical twin^8^.

### Example of calculating quantitative outputs

To demonstrate a quantitative result of the digital twin, an estimation of the parametric uncertainty of the stiffness parameters $k_1, k_2, k_3$ from Agent 4, was carried out using Agent 5. A uniform prior distribution ranging from $[30,000, \, 50,000]$N/m is set as the initial estimate for the prior distributions of the parameters $k_1, k_2, k_3$. Agent 5, Bayesian model calibration method, was then used to calibrate the analytical model by inferring the parameters $k_1, k_2, k_3$^8,52^, and the results are shown as quantitative and visual outputs in Fig. 1g. The discrepancy between the inferred (red line distributions) and the “ground truth” values (vertical black lines) in Fig. 1g is less than 200 N/m, which can be taken to be a reasonable result considering the range of the uniform prior distribution.

As the workflow progresses, the agents’ outputs are dynamically updated both in the knowledge graph and the relational database, and the results will be displayed via the user interface as shown in Fig. 2.

### Information management system

The information management system (IMS)^16^ coordinates all data, information and knowledge within the digital twin. All the knowledge of these digital assets is modeled through a structured, ontology-based approach. Rooted in the philosophical exploration of being, existence, and reality, the term *ontology* has been adapted in information technology to describe a structured framework of concepts used to model a particular domain of knowledge. In the context of this digital twin, the ontology functions as a foundational blueprint that defines the classification, attributes, and interrelationships of entities across all components of the digital twin. For the example structure described in this paper, a more detailed ontology, including geometrical and behavioral, is described in the authors’ previous work^53^. Note that, we have chosen to develop a custom domain ontology in Neo4j because they can carry rich contextual information, including timestamps, values, and other attributes, which are not natively supported in other ontology models.

In this digital twin, the IMS consists of a knowledge graph and two relational databases. The first relational database contains information on material properties, joints, sensors, actuators, loads, boundary conditions, and conditions of the test environment (e.g. Fig. 1d). The second relational database contains the sensor data recorded from the physical twin (e.g., Fig. 1f).

### Structure and time evolution of the knowledge graph

The knowledge graph was built using Neo4j^18^, and in this example is a property graph^41^ denoted as the tuple $G=\langle V, E, L, U, \ell , e, r \rangle$ where *V* is the set of entities (graph nodes), *E* is the set of relationships (graph edges), *L* the labels, *U* are property-value pairs and $\ell , e, r$ are mappings within the graph. Specifically, $\ell$ is the mapping that defines each of the labels in *L* to the elements in *V* and *E*. Then *e* maps the relationships (edges) *E* to a pair of entities (nodes) in *V*. Finally, *r* maps entities and relationships to property-value pairs defined in *U* — see Algorithm B in Fig. 5.

**Fig. 6:**
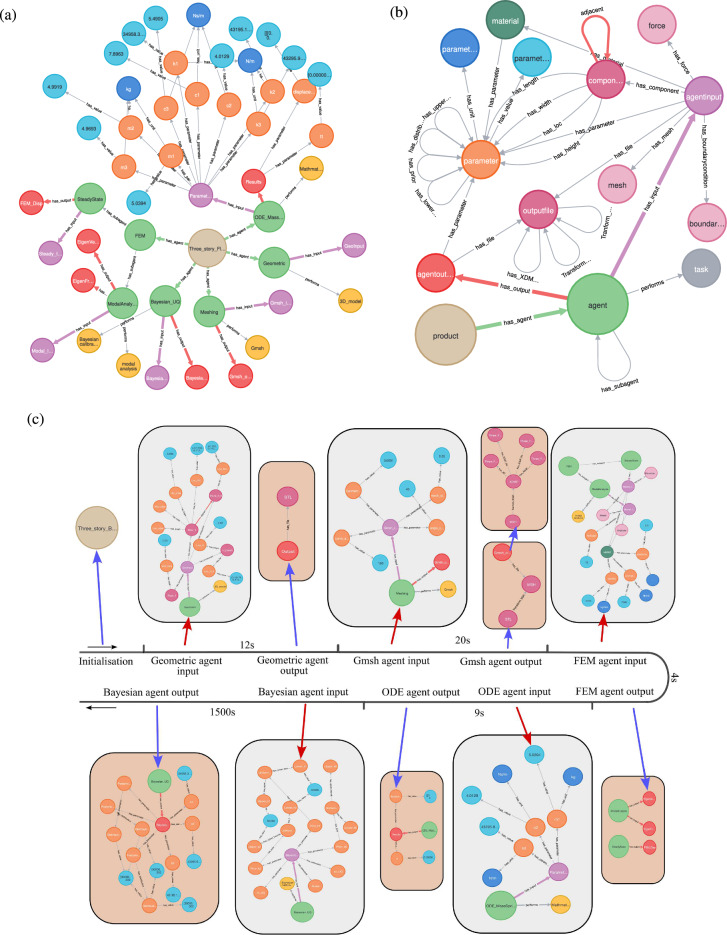
Examples of the knowledge graph structure, showing **a** the three-story building knowledge graph with the primary entity of the graph (Layer 0) as the sand color entity, and the agents as green entities — note a number of entities and relationships have been removed from this Figure to make it readable. **b** A meta-graph, indicating a generic graph structure for a generic agent, with a representative example of the associated entities and relationships. **c** Evolution of the knowledge graph; the KG is initialized by defining the Layer 0 node, and it will subsequently be updated for each of the agent inputs (indicated by red arrows) and outputs (indicated by blue arrows) to the KG; the associated computational time between agent inputs and outputs is indicated in seconds (s). Note only Layers 0 and 1 are color coded in this figure.

A section of the resulting graph built from Algorithm B is shown in Fig. 6a, where circles represent entities and the relationships are shown using labeled single-direction arrows — note that a number of entities and relationships have been removed from Fig. 6a to make it readable. The graph is centered around a single entity, denoted Layer 0 (e.g. $V_0$, the sand colored entity in Fig. 6) representing the top-level part of the hierarchy in the graph; for example, we can identify this as the self-model. Then each of the *N* agents are represented at Layer 1 and shown as green entities in Fig. 6. Other entities are considered to be in Layers 2, 3 or 4 depending on the graph structure. In particular, Layers 3 and 4, are where the child characteristics of Layer 2 are defined and are inherently bespoke due to the heterogeneous nature of the components within the specific digital twin.

To give context, a single generic agent structure is shown in detail in Fig. 6b, which is assumed to be part of a generic ‘product’ at Level 0. Here examples of the detailed possible relationships, including self-relations and double entity relations are shown. The metagraph depicts how a range of agent architectures can be represented in the graph. Each agent within the DT has defined inputs and outputs and performs a distinct task. These tasks are predetermined, and therefore fixed in the graph structure. In contrast, input/output parameters, files, or components created during the agents’ operation will be added to the graph dynamically as the workflow proceeds. An example of the evolution of the knowledge graph is shown in Fig. 6c.

A time-stamped recording approach is employed for new events occurring within the DT, which are stored as entities or relationships in the KG. These timestamps effectively reflect the computational efforts of associated agents. Each agent operates in two distinct modes: ‘on’ and ‘off’. In the ‘on’ mode, agents execute predefined computational functionalities, whereas in the ‘off’ mode, the KG is updated to include either input or output data. The computational durations of the five agents were recorded as 9 seconds, 12 seconds, 20 seconds, 4 seconds, and 1500 seconds (25 minutes), respectively, as shown in Fig. 6c.

### RAG query system design

The information management system includes a natural language querying function which in this case is enabled using RAG between the local knowledge graph and a LLM. LLMs have gained much attention since the launch of Chat-GPT in late 2022^43^. LLMs allow users to retrieve a comprehensive answer to a query (prompt), but the answer does not include information drawn from the specific domain defined by the user, especially from a specific application-based knowledge graph, or the most recent information (before the LLM was trained).

The user interface shown in Fig. 2 was built using a combination of HTML, CSS, and JavaScript. It contains a graph visualization powered by the D3.js library^54^. JavaScript is used to process user interactions, including fetching graph data from a server endpoint and rendering it using a force-directed graph layout. The nodes and edges of the graph are drawn with varying sizes and colors based on their labels and relationships. A custom centering force ensures that the Layers 0 & 1 node types (e.g. “product” and “agent”) are positioned strategically within the graph based on the assumption that the digital twin should be centered around the start node of the self-model.

In Fig. 2, the chatbox element is used to input natural language queries. The RAG is achieved by establishing a connection to the Neo4j graph database, facilitating semantic queries to the knowledge graph. Then the LangChain^55^ library is employed to query the graph using the natural language input from the user. Langchain processes the input into Cypher language, the underlying query language used to query the Neo4j graph database. If the Cypher query to the Neo4j graph fails to return a valid response, the system reverts to the OpenAI language model (GPT-4), querying the model via the OpenAI library using a chat-based completion approach. As a demonstration, the back-end log for the example shown in the top right side of Fig. 2, is shown below:
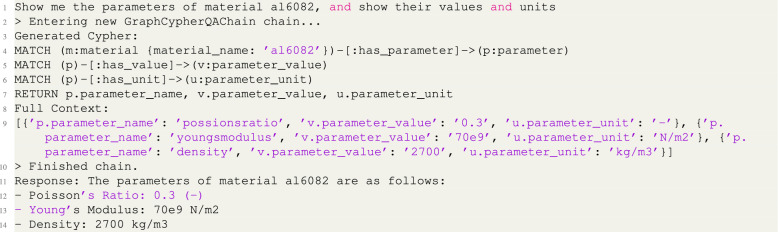


Notice that in the Full Context the parameter values are stored as key value pairs from *U*, for example for Poisson’s ratio as $'p.parameter\_name':\; 'possionsratio',\; 'v.parameter\_value':\;'0.3'$.

## Discussion

The digital twin architecture presented here enables the ‘assembly’ of a series of heterogeneous digital components into a workflow that can be used to build a self-model. To enable the modes of operation, and switches between them, it is important that agents can both collaborate and work autonomously. We have shown one, small-scale, example of how this can be achieved for the case of just a single workflow in offline mode, and from that the initial self-model is encoded in the form of a knowledge graph. In this small-scale example, the knowledge graph is fulfilling the role of both a graph database and a knowledge-based representation of the self-model. Relational databases are used to store other information relevant to the overall self-model.

Knowledge graphs can be highly effective for managing information, as we have shown, by designing a hierarchical architecture in which the digital twin self-model concept is represented as the Layer 0 node, and agents serve to provide functionalities at Layer 1 of the digital twin. Dynamic knowledge graphs can be used to create ‘digital threads’ which can significantly enhance the life-cycle management of assets^56^. This is because, the timestamped event information, stored in the property graph, allows users to trace back the activities of agents that occurred within the digital twin. The ultimate goal of using these types of knowledge graphs is to integrate more sophisticated functions such as knowledge manipulation and reasoning capabilities^12,42^.

The example chosen for this proof-of-concept work was quite small-scale, but we believe that the architecture could be applied to many other applications, including at scale. Here, the purpose of the digital twin is chosen purely to demonstrate how an agent-based architecture can be used to deliver a specific outcome. Other choices could be made depending on the specific context of the application of interest. In this paper, we define the purpose as creating a system of self-knowledge and enabling users to make queries via a user interface to access the stored information. To achieve this functionality, a sample workflow for the digital twin is developed that integrates geometric, material, and behavioral properties of the building structure in addition to a series of analytical & numerical methods that create predictions and estimate uncertainties.

The output of our chosen example was a measure of parameter uncertainty. Future online workflows could use newly measured data to update multi-fidelity prediction models and perform more sophisticated uncertainty quantification and propagation methods. The high-fidelity model (such as FEA in Agent 3) can also be used to update and re-calibrate the low-fidelity models (like ODE in Agent 4) once updated. By continuously incorporating new information, the digital twin predictions remain up-to-date, while historical data are preserved for future retrieval via a digital thread. It is possible that future DTs could have simultaneous online/offline connectivity modes and therefore be operating both physical and virtual environments at the same time. Also, we note that the example used here has significant similarities with building information management (BIM) systems which have also been explored in connection with digital twinning^57^.

However, it should also be noted that there are some limitations of the architecture proposed here. For example, by using agents in this manner, it will not always be easy (or even possible) to represent the coupling between physics that are not already contained in an individual agent. In particular, here we use a stepped workflow approach (similar to how most FEA software does multiphysics) that ignores the non-propagation aspects of the component parts of the overall model. For example, it could propagate the thermal stress but would not typically be able to propagate the thermal expansion/volume change.

Although not considered here, actions can be sent to ‘actuators’ (or other forms of action-taking mechanism acting on the physical twin) in the case that real-world actions are required. In the current example of the three-story building, one method of taking actions is provided by force from a vibration ‘shaker’ (shown in Figure 1 (e) attached to the three-story physical twin).

## Conclusion

In this paper, an agent-based digital twin architecture has been used to create a self-model of a small-scale three-story building. The architecture was based on a set of digital components, each managed by an agent, and used to create a self-model encoded in a knowledge graph. The agents can be orchestrated to perform a specific workflow or collaborate with a human user to perform requested tasks.

The workflow chosen here enabled both complex geometries and behaviors to be represented. In this case via a time-evolving dynamic assembly of the digital components, which also included uncertainty quantification of selected parameters within the digital twin. Four operational modes were defined, based on the combination of work and operational modes. The example shown here demonstrated an offline mode with agent-based orchestration to carry out a predefined workflow with five agents.

The information management system was coordinated using a dynamic knowledge graph that encoded the self-model. Users can visualize the graph representing the self-model via a web-based user interface. Natural language queries can be input by the human user, and a form of retrieval augmented generation was used to give response to the queries using both the local knowledge graph and a large language model.

## Data Availability

All code and data is available via: https://github.com/Digital-Twin-Operational-Platform/KG/tree/main/digitaltwin

## References

[1] Crawley, E. F. Intelligent structures for aerospace-a technology overview and assessment. *AIAA J.* **32**, 1689–1699 (1994).

[2] Guran, A. & Inman, D. J. *Intelligent Structures and Nonlinear Mechanics* (World Scientific).

[3] Chopra, I. & Sirohi, J. *Smart Structures Theory* Vol. 35 (Cambridge University Press, 2013).

[4] Salehi, H. & Burgueño, R. Emerging artificial intelligence methods in structural engineering. *Eng. Sruct.* **171**, 170–189 (2018).

[5] Tapeh, A. T. G. & Naser, M. Artificial intelligence, machine learning, and deep learning in structural engineering: a scientometrics review of trends and best practices. *Arch. Comput. Methods Eng.* **30**, 115–159 (2023).

[6] Kwiatkowski, R., Hu, Y., Chen, B. & Lipson, H. On the origins of self-modeling. arXiv preprint arXiv:2209.02010 (2022).

[7] Chen, B., Kwiatkowski, R., Vondrick, C. & Lipson, H. Fully body visual self-modeling of robot morphologies. *Sci. Robot.* **7**, eabn1944 (2022). 35857575 10.1126/scirobotics.abn1944

[8] Wagg, D. J., Worden, K., Barthorpe, R. J. & Gardner, P. Digital Twins: State-of-the-Art and Future Directions for Modeling and Simulation in Engineering Dynamics Applications. *ASCE-ASME J. Risk Uncert. Engrg. Sys. Part B Mech. Engrg.* **6**, 030901 (2020).

[9] Yue, Y. A world-self model towards understanding intelligence. *IEEE Access* **10**, 63034–63048 (2022).

[10] Bar, A., Zhou, G., Tran, D., Darrell, T. & LeCun, Y. Navigation world models. arXiv preprint arXiv:2412.03572 (2024).

[11] Akroyd, J., Mosbach, S., Bhave, A. & Kraft, M. Universal digital twin–a dynamic knowledge graph. *Data-Centric Eng.* **2**, e14 (2021).

[12] Bai, J. et al. A derived information framework for a dynamic knowledge graph and its application to smart cities. *Future Gener. Comput. Syst.* **152**, 112–126 (2024).

[13] Quek, H. Y. et al. Dynamic knowledge graph applications for augmented built environments through “the world avatar’’. *J. Build. Eng.* **91**, 109507 (2024).

[14] Hetherington, J. & West, M. The pathway towards an information management framework-a ‘commons’ for digital built britain. CDBB white paper (2020). https://www.cdbb.cam.ac.uk/files/the_pathway_towards_an_imf.pdf.

[15] Sacks, R., Brilakis, I., Pikas, E., Xie, H. S. & Girolami, M. Construction with digital twin information systems. *Data-Centric Eng.* **1**, e14 (2020).

[16] Singh, S. et al. Towards information management framework for digital twin in aircraft manufacturing. *Procedia CIRP* **96**, 163–168 (2021).

[17] Correia, J. B., Abel, M. & Becker, K. Data management in digital twins: a systematic literature review. *Knowl. Inf. Syst.* **65**, 3165–3196 (2023).

[18] Bratanic, T. *Graph Algorithms for Data Science: with Examples in Neo4j* (Simon and Schuster, 2024).

[19] Clemen, T. *et al.* Multi-agent systems and digital twins for smarter cities. In *Proceedings of the 2021 ACM SIGSIM conference on principles of advanced discrete simulation*, 45–55 (2021).

[20] Caldarelli, G. et al. The role of complexity for digital twins of cities. *Nat. Comput. Sci.* **3**(5), 374–81 (2023). 38177836 10.1038/s43588-023-00431-4

[21] Francisco, A., Mohammadi, N. & Taylor, J. E. Smart city digital twin-enabled energy management: Toward real-time urban building energy benchmarking. *J. Manag. Eng.* **36**(2), 04019045 (2020).

[22] Lu, Q. et al. Developing a digital twin at building and city levels: Case study of west Cambridge campus. *J. Manag. Eng.* **36**, 05020004 (2020).

[23] Wagg, D. J. et al. The philosophical foundations of digital twinning. *Data-Centric Eng.* **6**, e12 (2025).

[24] Metzinger, T. *Being No One: The Self-model Theory of Subjectivity* (MIT Press, 2004).

[25] Zheng, X. et al. A quality-oriented digital twin modelling method for manufacturing processes based on a multi-agent architecture. *Procedia Manuf.* **51**, 309–315 (2020).

[26] Croatti, A., Gabellini, M., Montagna, S. & Ricci, A. On the integration of agents and digital twins in healthcare. *J. Med. Syst.* **44**, 1–8 (2020). 10.1007/s10916-020-01623-5 PMC7399680 32748066

[27] Vrabič, R., Erkoyuncu, J. A., Farsi, M. & Ariansyah, D. An intelligent agent-based architecture for resilient digital twins in manufacturing. *CIRP Ann.* **70**, 349–352 (2021).

[28] Marah, H. & Challenger, M. Madtwin: a framework for multi-agent digital twin development: smart warehouse case study. *Ann. Math. Artif. Intell.* **92**, 975–1005 (2024).

[29] Wooldridge, M. Intelligent agents. In *Multiagent Systems: A Modern Approach to Distributed Artificial Intelligence* 27–73 (MIT Press, 1999).

[30] Wooldridge, M. *An Introduction to Multiagent Systems* (Wiley, 2009).

[31] Russell, S. & Norvig, P. *Artificial Intelligence: A Modern Approach* (Pearson, 2010).

[32] Abar, S., Theodoropoulos, G. K., Lemarinier, P. & O’Hare, G. M. Agent based modelling and simulation tools: A review of the state-of-art software. *Comput. Sci. Rev.* **24**, 13–33 (2017).

[33] Dziomin, U., Kabysh, A., Stetter, R. & Golovko, V. A multi-agent reinforcement learning approach for the efficient control of mobile robots. In *Advances in Intelligent Robotics and Collaborative Automation* 123–145 (River Publishers, 2022).

[34] Wang, L. et al. A survey on large language model based autonomous agents. *Front. Comput. Sci.* **18**, 186345 (2024).

[35] Acharya, D. B., Kuppan, K. & Divya, B. Autonomous intelligence for complex goals-a comprehensive survey. *IEEE Access* **13**, 18912–18936 (2025).

[36] Kapteyn, M. G., Pretorius, J. V. R. & Willcox, K. E. A probabilistic graphical model foundation for enabling predictive digital twins at scale. *Nature* **1**, 337–347 (2021). 10.1038/s43588-021-00069-0 38217207

[37] Tezzele, M., Carr, S., Topcu, U. & Willcox, K. E. Adaptive planning for risk-aware predictive digital twins. arXiv preprint arXiv:2407.20490 (2024).

[38] Allemang, D. & Hendler, J. *Semantic Web for the Working Ontologist: Effective Modeling in RDFS and OWL* (Elsevier, 2011).

[39] Fensel, D. et al. *Knowledge Graphs* (Springer, 2020).

[40] Bergman, M. K., Bergman, M. K. & Lagerstrom-Fife,. *Knowledge Representation Practionary* (Springer, 2018).

[41] Hogan, A. et al. Knowledge graphs. *ACM Comput. Surv. (Csur)* **54**, 1–37 (2021).

[42] Zhou, B. et al. Semantic-aware event link reasoning over industrial knowledge graph embedding time series data. *Int. J. Prod. Res.* **61**(12), 4117–34 (2022).

[43] Teubner, T., Flath, C. M., Weinhardt, C., van der Aalst, W. & Hinz, O. Welcome to the era of chatgpt et al. the prospects of large language models. *Bus. Inf. Syst. Eng.* **65**, 95–101 (2023).

[44] Wang, P. On defining artificial intelligence. *J. Artif. Gen. Intell.* **10**, 1–37 (2019).

[45] Ieva, S. et al. A retrieval-augmented generation approach for data-driven energy infrastructure digital twins. *Smart Cities* **7**, 3095–3120 (2024).

[46] Xia, Y., Xiao, Z., Jazdi, N. & Weyrich, M. Generation of asset administration shell with large language model agents: Interoperability in digital twins with semantic node. arXiv preprint arXiv:2403.17209 (2024).

[47] Luck, M. & Aylett, R. Applying artificial intelligence to virtual reality: Intelligent virtual environments. *Appl. Artif. Intell.* **14**, 3–32 (2000).

[48] 3D Systems, Inc., “stl file format specification,” 1989. [online]. https://web.archive.org/web/20170105062756/, http://www.fabbers.com/tech/STL_Format

[49] Geuzaine, C. & Remacle, J.-F. Gmsh: A 3-d finite element mesh generator with built-in pre-and post-processing facilities. *Int. J. Numer. Methods Eng.* **79**, 1309–1331 (2009).

[50] Geveci, B., Moreland, K. & Ahrens, J. Xdmf: extensible data model and format. retrieved from. https://www.xdmf.org/.

[51] Baratta, I. A. *et al.* Dolfinx: the next generation fenics problem solving environment (2023).

[52] Kennedy, M. C. & O’Hagan, A. Bayesian calibration of computer models. *J. R. Stat. Soc.: Ser. B (Stat. Methodol.)* **63**, 425–464 (2001).

[53] Shen, X., Devaraja, P., Wagg, D. & Bonney, M. S. A structured knowledge graph for a geometric and behavioral digital twin in the context of modal testing. In *IMAC, A Conference and Exposition on Structural Dynamics*, 5–14 (Springer, 2024).

[54] Bostock, M., Ogievetsky, V. & Heer, J. data-driven documents. *IEEE Trans. Vis. Comput. Gr.* **17**, 2301–2309 (2011). 10.1109/TVCG.2011.185 22034350

[55] Topsakal, O. & Akinci, T. C. Creating large language model applications utilizing langchain: A primer on developing llm apps fast. In *International Conference on Applied Engineering and Natural Sciences* **1**, 1050–1056 (2023).

[56] Singh, V. & Willcox, K. E. Engineering design with digital thread. *AIAA J.* **56**, 4515–4528 (2018).

[57] Sepasgozar, S. M. et al. BIM and digital twin for developing convergence technologies as future of digital construction. *Buildings* **13**, 441 (2023).

